# Haemodynamic effects of the flavonoid quercetin in rats revisited

**DOI:** 10.1111/bph.14955

**Published:** 2020-02-03

**Authors:** Misha F. Vrolijk, Helma van Essen, Antoon Opperhuizen, Aalt Bast, Ben J. Janssen

**Affiliations:** ^1^ Department of Pharmacology & Toxicology, Faculty of Health, Medicine and Life Sciences Maastricht University Maastricht The Netherlands; ^2^ Faculty of Science and Engineering Maastricht University Campus Venlo Venlo The Netherlands; ^3^ Office for Risk Assessment andResearch (BuRO) Netherlands Food and Consumer Product Safety Authority (NVWA) Utrecht The Netherlands

## Abstract

**Background and Purpose:**

The flavonoid quercetin increased the *in vitro* potency of the α_1_‐antagonist tamsulosin to reduce phenylephrine‐dependent arterial contractions by 10‐fold. To examine if this supplement–drug interaction luxates hypotensive and orthostatic events *in vivo*, several set of studies were conducted in spontaneously hypertensive (SHR) and normotensive (Wistar Kyoto [WKY]) rats.

**Experimental Approach:**

First, in rats pretreated with quercetin or its vehicle, responses to phenylephrine and tamsulosin were examined. Second, tamsulosin‐induced changes in renal, mesenteric, hindquarter and carotid conductance were compared in quercetin‐ and vehicle‐treated rats instrumented with Doppler flow probes. Animals were also placed on a tilt table to record regional haemodynamic changes to orthostatic challenges. Third, adult SHR were instrumented with telemeters to measure 24‐hr patterns of BP. Recordings were made before and during a 5‐week oral treatment of quercetin. Finally, pre‐hypertensive SHR were treated with quercetin from 4 to 8 weeks of age and arterial pressure was measured at 8 and 12 weeks.

**Key Results:**

Pretreatment with quercetin did not influence the responses to phenylephrine and tamsulosin, in neither WKY nor SHR. While tamsulosin treatment and tilting lowered BP and increased conductance in all vascular beds, effect size was not influenced by pretreatment with quercetin. Prolonged treatment with quercetin, in either prehypertensive SHR or adult SHR with established hypertension did not lower BP.

**Conclusions and Implications:**

Cumulatively, these data demonstrate that quercetin does not amplify haemodynamic effects of tamsulosin or tilting *in vivo* in rats and has no effect on BP development in SHR.

**Abbreviations**
HR: heart rate
MAP: mean arterial pressure
SHR: spontaneously hypertensive rats
WKY: Wistar Kyoto

**What is already known**

- Tamsulosin may evoke orthostatic hypotension by blocking vascular α‐adrenoceptors.
- Quercetin amplifies the *in vitro* vasorelaxing potency of the α‐antagonist tamsulosin.

**What this study adds**

- Quercetin does not attenuate the phenylephrine‐induced hypertensive responses *in vivo* in rats.
- Quercetin does not amplify the tamsulosin‐induced hypotensive responses *in vivo* in rats.

**What is the clinical significance**

- Hypotensive dynamic interactions between the supplement quercetin and tamsulosin are unlikely.

## 1 INTRODUCTION

Epidemiological studies have revealed inverse associations between the intake of dietary flavonoids and mortality from cardiovascular diseases (Geleijnse, Launer, Van der Kuip, Hofman, & Witteman, 2002; Hertog, Feskens, Hollman, Katan, & Kromhout, 1993; Knekt, Jarvinen, Reunanen, & Maatela, 1996; McCullough et al., 2012; Mink et al., 2007; Yochum, Kushi, Meyer, & Folsom, 1999). https://www.guidetopharmacology.org/GRAC/LigandDisplayForward?ligandId=5346 is the most abundant flavonoid in fruits and vegetables and is the best studied flavonoid. Prominent effects attributed to quercetin include antioxidant, anti‐inflammatory, anti‐allergic, anti‐carcinogenic and antihypertensive, as well as anti‐atherogenic activities (Boots, Drent, de Boer, Bast, & Haenen, 2011; Kleemann et al., 2011; Patel et al., 2018). Quercetin is therefore regarded to have potentially beneficial health effects and is widely available as a food supplement (Boots, Haenen, & Bast, 2008).

Duarte et al. (2001) reported that oral intake of quercetin (10 mg·kg^−1^) lowers arterial BP in spontaneously hypertensive rats (SHR). The same treatment regimen also attenuated l‐NAME‐induced hypertension in rats (Perez‐Vizcaino et al., 2002). Studies on isolated vessels isolated from these rat models suggested that the vasodilator effect was inversely proportional to vessel size and occurred especially in the mesenteric vascular bed (Perez‐Vizcaino et al., 2002). While other studies have explored the antihypertensive effects of quercetin further, controversy exists about the potential mechanisms involved, as well the magnitude of the effect and the potential clinical relevance (Carlstrom et al., 2007; Larson, Symons, & Jalili, 2012; Monteiro, Franca‐Silva, Alves, Porpino, & Braga, 2012; Sanchez et al., 2006). This is possibly due to publication bias (Serban et al., 2016), since negative studies on quercetin have been rarely published. Recently, we discovered that in isolated mesenteric arteries, quercetin increased the potency of https://www.guidetopharmacology.org/GRAC/LigandDisplayForward?ligandId=488 to inhibit https://www.guidetopharmacology.org/GRAC/LigandDisplayForward?ligandId=485‐induced contractions by a factor >10 (Vrolijk et al., 2015). Tamsulosin is prescribed to male patients to facilitate urination by blocking α_1_‐https://www.guidetopharmacology.org/GRAC/FamilyDisplayForward?familyId=4 in the prostate and neck of the bladder. Blockade of vascular α_1_‐adrenoceptors has been associated with the occurrence of orthostatic (postural) hypotensive (Akiyama, Hora, Yamagishi, & Kitazawa, 2002; Noble et al., 1997). The present study was designed to explore if the supplement–drug interaction between quercetin and tamsulosin would also occur *in vivo* in rats. To investigate the possible haemodynamic mechanisms involved, four series of experiments were performed assessing acute as well as chronic effects of oral and intravenously administered quercetin in both SHR and Wistar Kyoto (WKY) rats.

## 2 METHODS

### 2.1 Animals, ethical statement and husbandry

All animal procedures were carried out with the approval of the Animal Ethics Committee of the Maastricht University and are in accordance with the editorial guidelines on experimental design and analysis in pharmacology (Curtis et al., 2018). The experimental studies were conducted in accordance to those published in the European Directive 2010/63/EU or earlier versions. Animal studies are reported in compliance with the ARRIVE guidelines (Kilkenny, Browne, Cuthill, Emerson, & Altman, 2010) and *with the editorial on reporting animal studies* (McGrath & Lilley, 2015). Group sizes were a priori determined using mandatory power calculations based on the outcome of comparable previous studies.

Series of experiments were performed in inbred hypertensive SHR/NCrl and normotensive WKY rats/NCrl. These rat models have been used extensively to examine the antihypertensive potency of agents *in vivo* (Doris, 2017). All rats were delivered by a commercial breeder (Charles River, Germany). Unless specified otherwise, animals were housed in groups in standard cages, in a temperature‐controlled (22 ± 2°C) room, with lights on from 07:00 a.m. to 07:00 p.m. and had free access to food and water throughout. Various experimental interventions required single housing of the animals. Details are outlined in the various studies below.

### 2.2 Experimental procedures and surgery

#### 2.2.1 Study 1: Does quercetin amplify the α_1_‐adrenoceptor blocking properties of tamsulosin *in vivo*?

Since SHR are considered to have elevated sympathetic tone and are more sensitive to effects of α‐antagonists, the first series of studies were conducted in SHR as well as WKY rats. Studies were done in eight SHR/NCrl (4 females + 4 males) and eight WKY rats/NCrl (4 females + 4 males). The male and female rats were housed in the same room but not in the same cage. The menstrual cycle was not determined in the females. All rats received pre‐operatively https://www.guidetopharmacology.org/GRAC/LigandDisplayForward?ligandId=1670 (0.01 mg·kg^−1^, s.c.). Rats were placed in a box and anaesthesia was induced by inhalation of https://www.guidetopharmacology.org/GRAC/LigandDisplayForward?ligandId=2505 (4%). Anaesthesia was maintained by inhalation of isoflurane (1.5–2.5%) through a nose cone. A catheter was inserted into the lower abdominal aorta via the femoral artery. A second line was inserted into the femoral vein. Both catheters were tunnelled subcutaneously to the neck of the rats, filled with heparinized saline (5 IU·ml^−1^) and closed with a metal plug. After regaining consciousness, analgesia was maintained by subcutaneous injection of https://www.guidetopharmacology.org/GRAC/LigandDisplayForward?ligandId=7141 (5 mg·kg^−1^). This injection was repeated 24 hr later. Rats were now housed individually and allowed to recover from surgery for 72 hr. The instrumented rats were subjected to the following two experimental protocols.

##### Protocol 1A

Four SHR/NCrl (2 females + 2 males) and four WKY rats/NCrl (2 females + 2 males) were dosed twice with quercetin by administering the agent (5 mg·kg^−1^, i.v., max. 300 μl): once immediately after placement of the catheters and once 48 hr after surgery. The dose was based on studies that were conducted before and the route of administration was chosen to overcome the low oral bioavailability of quercetin making sure that the system was loaded with the supplement (Duarte et al., 2001; Jalili et al., 2006; Li et al., 2009; Perez‐Vizcaino et al., 2002; Sanchez et al., 2006). Control rats were injected with vehicle solution (cyclodextrin 15% w/v in saline). Twenty‐four hours after the second loading dose, rats were placed in a small cage containing some of the bedding of the original home cage to limit stress reactions. The arterial and venous lines were extended to record arterial BP and to allow injections of drug solutions. Haemodynamics were allowed to stabilize for at least 1 hr before the actual recordings started. First, baseline BP was recorded for 30 min. Then, BP changes to four incremental dosages of phenylephrine (0.1, 0.3, 1.0 and 3.0 μg·kg^−1^, i.v.) were determined. The volume that was maximally injected intravenously was 250 μl (50 μl of the phenylephrine solution followed by 200 μl of saline to flush the line). The four injections were given in a 15‐min interval period. Next, quercetin or its vehicle was administered intravenously, in the same dose and volume as before, after which the baseline BP was recorded for 15 min and the phenylephrine challenges were repeated. Then, tamsulosin (3 μg·kg^−1^, i.v., max. 300 μl) was administered and phenylephrine challenges were repeated at 10, 40 and 120 min after the tamsulosin injection. After completion of this experiment, catheters were flushed with heparinized saline and rats were allowed to rest in their home cage for at least additional 48 hr.

##### Protocol 1B

Two days later, the same rats were connected to the measuring equipment as described in protocol 1A. After stabilization of haemodynamics, baseline BP was recorded for 30 min. Then, quercetin (5 mg·kg^−1^, i.v.) or the vehicle solution was administered again and BP was recorded for another 15 min. Thereafter, BP changes were obtained in response to cumulatively increasing intravenous dosages of tamsulosin (0.3, 1.0, 3.0, 10, 30 and 100 μg·kg^−1^) given at 10‐ to 12‐min intervals. Injection volumes never exceeded 200 μl. At the end of the experiment the rats received a terminal dose of anaesthetic (i.v.; https://www.guidetopharmacology.org/GRAC/LigandDisplayForward?ligandId=5480 200 mg·kg^−1^).

#### 2.2.2 Study 2: Regional vascular effects of quercetin in the absence and presence of tamsulosin

As it was reported that quercetin preferentially dilated in rat mesenteric arteries (Perez‐Vizcaino et al., 2002), this study was designed to explore which vascular beds were targeted by the food additive and if the interaction with tamsulosin would unmask effects of quercetin in other vascular beds in adult male and female WKY rats (RRID:RGD_61103).

All rats were pretreated with 0.01 mg·kg^−1^ of buprenorphine subcutaneously and anaesthesia was induced and maintained as described in protocol 1A. When pedal withdrawal reflexes were absent, a ventral midline abdominal incision was made, intestines were retracted and target vessels were isolated by blunt dissection. Miniature pulsed Doppler flow probes were fixed around the left renal artery, the superior mesenteric artery, the distal abdominal aorta and the left common carotid artery to measure renal, mesenteric, hindquarter and carotid blood flow, respectively, as described previously (Janssen, van Essen, Struyker Boudier, & Smits, 1989). The wires of the probes were exteriorized and the peritoneal cavity was closed with sutures to limit hypothermia and fluid loss. Catheters were inserted in the femoral artery and vein to facilitate the recording of arterial pressure and injections of agents, respectively. Next, while maintaining the isoflurane anaesthesia, rats were secured on a tilt table. Wires and catheters were connected and body temperature was kept at 37°C via a servo‐controlled system with the sensor probe placed in the rectum of the rat. The pressure sensor was placed at the level of the heart of the animal. The current set‐up has been drawn in Figure S2. In this way, haemodynamic responses were recorded during orthostatic challenges (45° head‐up) of 2‐min duration. The potential of such a tilt protocol has been published before by Akiyama, Hora, Yamagishi, and Kitazawa(2002) who compared orthostatic responses with various α‐adrenoceptor antagonists in rats.

The following protocol was carried out: ‐ Baseline haemodynamics were recorded for 15 min. Then the rats received either (5 mg·kg^−1^, i.v.) quercetin (*n* = 13, mean body weight ± *SD*, 263 ± 57 g) or its vehicle cyclodextrin (*n* = 10, 259 ± 55 g). Recordings were made for another 10 min. Then, tamsulosin (3.0 μg·kg^−1^, i.v.) was given and recordings were continued for additional 30 min. The preparation was subjected to orthostatic challenges before and after injection of the quercetin/vehicle solution and at 3, 10, 20 and 30 min after tamsulosin was applied. At the end of the study, the preparation was terminated by an intravenous injection of pentobarbital (200 mg·kg^−1^).

#### 2.2.3 Study 3: BP effects of oral administration of quercetin in SHR equipped with telemetric devices

Twelve male adult SHR (age 14–16 weeks, 280–320 g) were instrumented with implants (PA‐C40, Data Sciences International, St. Paul, MN, USA) to record arterial BP by telemetry. Anaesthesia was induced by isoflurane and perioperative analgesia was maintained with buprenorphine. The catheter was inserted in the abdominal aorta with its tip just below the renal arteries and the probe was placed in the peritoneal cavity as sutured the ventral abdominal muscle wall. The abdomen and skin were closed in layers with resorbable sutures. The animals were allowed to recover from surgery for 10–14 days. Animals were single housed now because the telemetric devices do not allow acquisition of multiple signals from one cage.

The following protocol was conducted:‐ Baseline BP, heart rate (HR) and locomotor activity were measured during three full 24‐hr cycles every week for a period of 6 weeks. The first week of this period (week 0) served as the baseline period. Next, from weeks 1 to 5, one group of SHR (*n* = 6, body weight 305 ± 23 g) received standard food to which quercetin was added. By measuring food consumption, the approximate daily oral intake of quercetin was estimated to be about 10 mg·kg^−1^. The SHR control group (*n* = 6, 294 ± 15 g) received the standard food only.

#### 2.2.4 Study 4: Effects of transient treatment on BP and renal haemodynamics in pre‐hypertensive SHR

Four‐week‐old male SHR/NCrl (*n* = 12) were treated with quercetin (1 g·kg^−1^) mixed through the standard food from weeks 4 to 8 of age. Control SHR (*n* = 12) received normal food. At the age of 8 weeks, BP was measured in conscious conditions by the same method as described in study 1. After these measurements, under isoflurane anaesthesia, renal blood flow was directly measured with a flow probe (Transonic Systems Inc., VB series 0.5 mm, Ithaca, NY, USA). Rats were killed by exsanguination and organs were harvested for later analysis. The same procedures were repeated in a subgroup of SHR (*n* = 6) that was retained until 12 weeks of age after the transient challenge with quercetin. Data were compared with those obtained in historic non‐treated controls (Heijnen, Van Essen, Schalkwijk, Janssen, & Struijker‐Boudier, 2014). Blood plasma samples were frozen and prepared for the later analysis of quercetin levels with the aid of a 6550 iFunnel Q‐TOF LC/MS (Agilent Technologies) similarly as described by Yang et al. (2016).

### 2.3 Data and statistical analysis

In Studies 1, 2 and 4, arterial BP was measured via fluid filled catheters that were coupled to a pressure transducer. Signals were digitally sampled (at >1 KHz) and processed by software (IDEEQ) developed by Maastricht Instruments BV (Maastricht University, The Netherlands) to extract mean arterial pressure (MAP) and HR on a beat‐to‐beat basis. In study 2, wires from the flow probes were connected to a Doppler flow meter (Crystal Biotech, Holliston, MA, USA) and processed by the same IDEEQ system in a comparable way.

In the protocols of study 1, baseline variables were obtained by averaging MAP and HR over at least 30‐min periods. Peak responses to phenylephrine were calculated as well as average 5‐min values of MAP and HR before any new injection was given. Peak responses of MAP following the incremental phenylephrine injections were averaged to acquire a cumulative index for the sensitivity to phenylephrine in each condition.

In study 2, baseline values of MAP, HR and regional blood flows were determined when the preparation had stabilized. Responses to drug injections were measured as mean values averaged over 5–10 min after injection. Responses to the 2‐min orthostatic tilt challenge were examined at 5, 10, 15, 30, 60, and 120 s after the stimulus. Regional conductance was derived by dividing the flow values by the pressure values.

In the telemetry study (#3), signal samples of 10‐s duration were taken every 5 min for three consecutive 24‐hr periods every week, including arbitrary units for locomotor activity. MAP and HR were derived from the waveform signal using the Dataquest ART System (Data Science, Inc.). Values of MAP, HR and locomotor activity were averaged over 1‐hr periods. The 24‐hr cycle was constructed by averaging the hourly over the three consecutive days.

In study 4, baseline parameters were obtained by averaging MAP and HR over at least 30‐min periods. Baseline values were obtained over a 15‐min period when the animal was under anaesthesia and the renal flow probe was in place.

In studies 1 and 2, a two‐way ANOVA was applied to examine the potential effects of treatment (quercetin vs. vehicle) and strain (WKY rats vs. SHR) on the haemodynamic parameters collected. In study 3, a two‐way ANOVA was applied to delineate the effect of treatment (quercetin vs. control) and time (week 0 vs. week 5). The post hoc tests were conducted only if F in ANOVA achieved *P* < .05 and there was no significant variance inhomogeneity. In Study 4, between‐group differences (quercetin vs. control) were only compared at one time point and unpaired Student's *t*‐tests were applied. Sample sizes subjected to statistical analysis included at least 6 animals per group (*n* = 6), where n = number of independent values. All statistical analyses were performed with GraphPad Prism 5.02 (GraphPad Prism, RRID:SCR_002798; GraphPad Software Inc., La Jolla, CA, USA). The data and statistical analysis comply with the recommendations of the *British Journal of Pharmacology* on experimental design and analysis in pharmacology.

#### 2.3.1 Drugs

Quercetin, phenylephrine, tamsulosin, and cyclodextrin were purchased from Sigma‐Aldrich. The specific use of these drugs is specified in detail in each protocol above. Buprenorphine hydrochloride (Buprecare) and isoflurane (IsoFlo) were obtained from AST Farma, Oudewater, The Netherlands. Carprofen (Norocarp) was obtained from Norbrook Laboratories, Newry, UK.

### 2.4 Nomenclature of targets and ligands

Key protein targets and ligands in this article are hyperlinked to corresponding entries in http://www.guidetopharmacology.org, the common portal for data from the IUPHAR/BPS Guide to PHARMACOLOGY (Harding et al., 2018), and are permanently archived in the Concise Guide to PHARMACOLOGY 2019/20 (Alexander et al., 2019).

## 3 RESULTS

### 3.1 Study 1: Does quercetin amplify the α_1_‐adrenoceptor antagonist action of tamsulosin *in vivo*?

#### 3.1.1 Baseline characteristics

Body weight, baseline BP and HR were comparable in vehicle‐ and quercetin‐pretreated SHR and WKY rats during the days that rats were subjected to the haemodynamic measurements in protocols 1A and 1B. As expected, BP was higher in SHR than in WKY rats. These data can be found in Figure S1.

##### Protocol 1A

**Figure 1:**
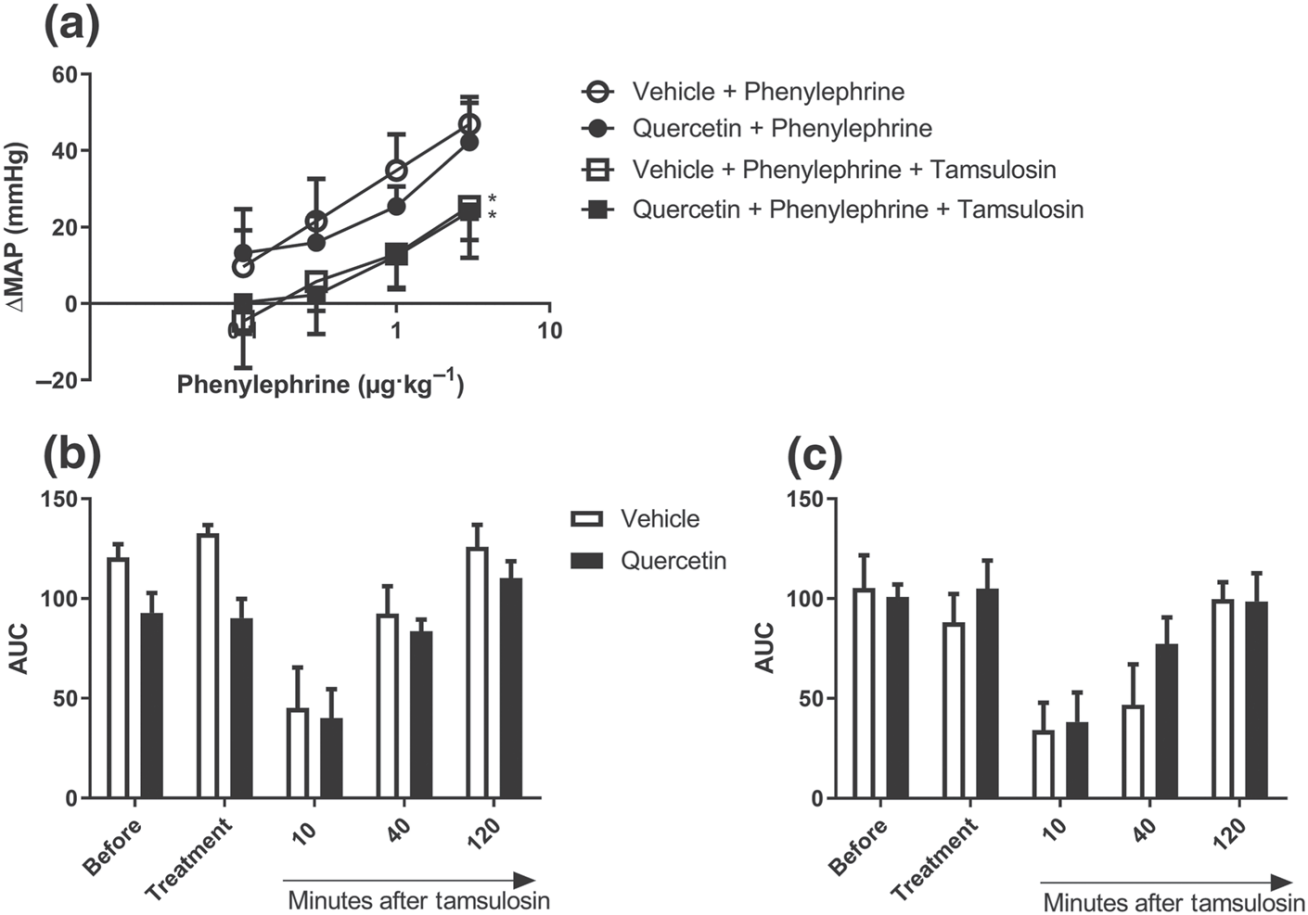
Comparison of phenylephrine‐induced pressor responses as recorded in unanaesthetized normotensive Wistar Kyoto rats (*n* = 8) and spontaneously hypertensive rats (*n* = 8) in vehicle‐ and quercetin‐treated animals. (a) The changes in mean arterial pressure (MAP) to intravenous bolus injections of phenylephrine before and after the intravenous administration of the α_1_‐antagonist tamsulosin. Tamsulosin attenuated, significantly and equipotently, the pressor responses to phenylephrine in both treatment arms. (b) Wistar Kyoto rats (*n* = 8) and (c) spontaneously hypertensive rats (*n* = 8) compare the averaged phenylephrine‐induced increments in MAP (averaged over the four injections) as shown in (a) and expressed as the cumulative AUC, before and after the intravenous injection of the vehicle or quercetin as well as at 10, 40 and 120 min after intravenous tamsulosin treatment. In both rat strains, blood pressure responses to α‐adrenoceptor stimulation or α‐adrenoceptor blockade were very comparable and not influenced by treatment of quercetin

Injections of phenylephrine induced a dose‐dependent increase in BP in all rats (Figure 1a). The magnitude of the pressor responses to phenylephrine was not different in vehicle‐ or quercetin‐pretreated animals. Intravenous administration of tamsulosin (3 μg·kg^−1^) attenuated the BP increments evoked by phenylephrine and resulted in a significant rightward shift of the pressor response curves. The extent of this rightward shift was not different between vehicle‐ and quercetin‐pretreated animals. As shown in Figure 1b,c, in WKY rats as well as SHR, cumulative phenylephrine‐induced pressor effects were time‐dependently inhibited by tamsulosin with the effect of tamsulosin wearing off at 40 min after injection. The duration of the blockade by tamsulosin was not different between vehicle‐ and quercetin‐pretreated rats.

##### Protocol 1B: The combined effect of a single dose of quercetin and cumulative doses of tamsulosin on haemodynamics

**Figure 2:**
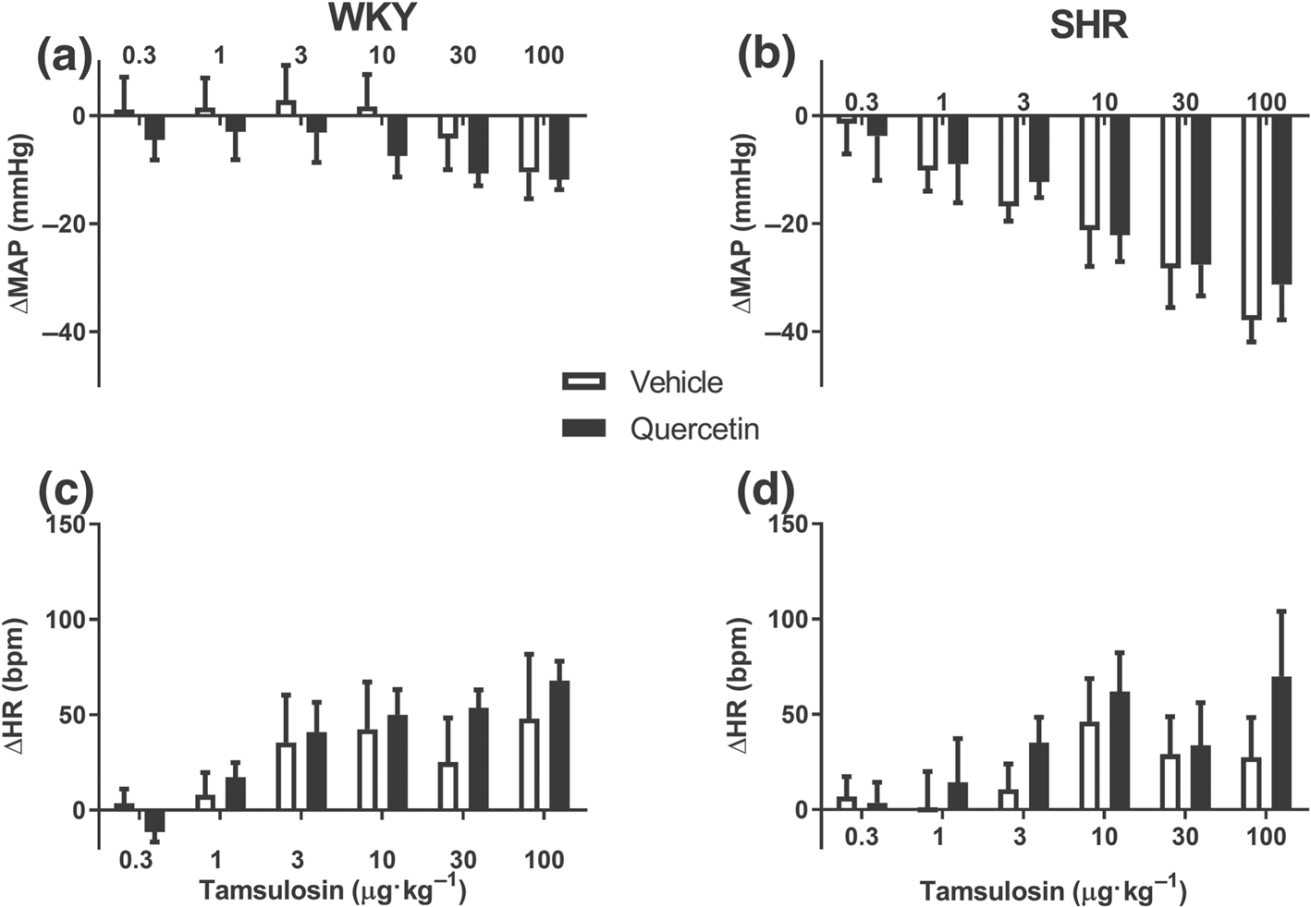
Comparison of the cumulative tamsulosin‐induced depressor responses as recorded in conscious normotensive Wistar Kyoto rats (WKY, *n* = 8) and spontaneously hypertensive rats (SHR, *n* = 8) in vehicle‐ and quercetin‐treated animals. (a, b) The changes in mean arterial pressure (MAP) in both strains. (c, d) The associated changes in heart rate (HR). While absolute depressor responses to tamsulosin were significantly greater in SHR than in WKY, blood pressure responses to α‐adrenoceptor blockade were not very different between vehicle‐ and quercetin‐treated rats

In vehicle‐pretreated unanaesthetized rats, dose–response curves of tamsulosin (0.3–100 μg·kg^−1^) mildly reduced BP in WKY rats (maximal effect −10 mmHg, Figure 2a), whereas potent reductions were observed in unanaesthetized SHR (maximal effect −40 mmHg, Figure 2b). HR increased dose dependently following tamsulosin injections in both SHR and WKY rats (Figure 2c,d). The magnitude of these changes in HR was not different for vehicle‐ or quercetin‐pretreated rats.

### 3.2 Study 2: Regional vascular effects of quercetin in the absence and presence of tamsulosin

**Figure 3:**
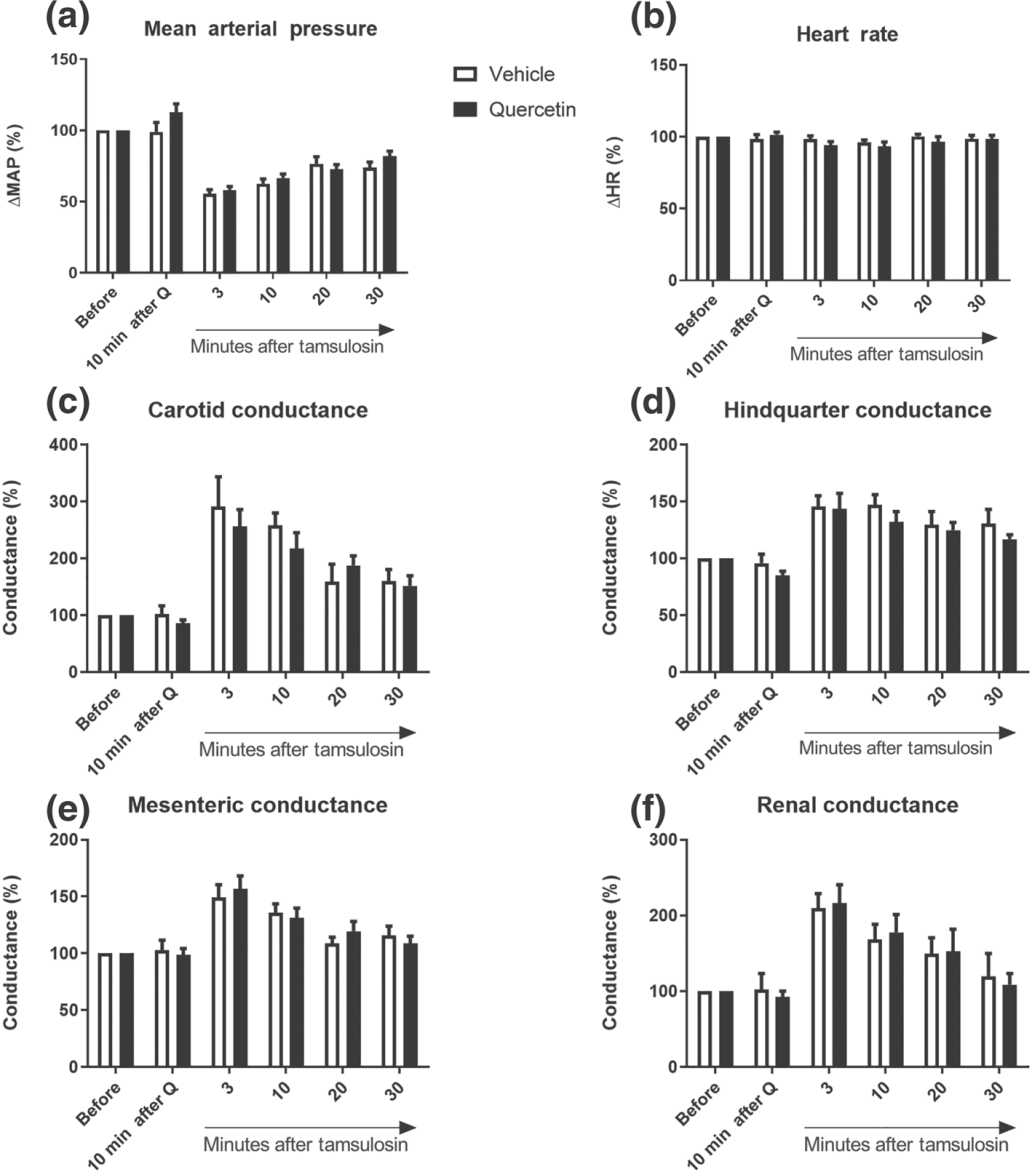
Comparison of the regional haemodynamic changes (mean arterial pressure (MAP; Figure 3a) and heart rate (HR; Figure 3b)) induced by acute intravenous injection of quercetin (*n* = 8) or its vehicle solution (*n* = 8) as recorded in isoflurane anaesthetized Wistar Kyoto rats instrumented with Doppler flow probes to record simultaneously blood velocity changes as observed in the four indicated vascular beds (carotid conductance (Figure 3c), hindquarter conductance (Figure 3d), mesenteric conductance (Figure 3e) and renal conductance (Figure 3f)). Data on tamsulosin‐induced haemodynamic changes were taken at 3, 10, 20 and 30 min after injection of the α_1_‐antagonist. Compared with vehicle treatment, quercetin treatment did not amplify tamsulosin‐induced changes in any of the vascular beds. Absolute baseline values (±*SD*) of MAP and HR under anaesthesia were not significantly different, being 70 ± 11 mmHg and 311 ± 34 bpm in the vehicle‐treated (*n* = 13) and 78 ± 11 mmHg and 325 ± 28 bpm in the quercetin‐treated (*n* = 10) rats, respectively

**Figure 4:**
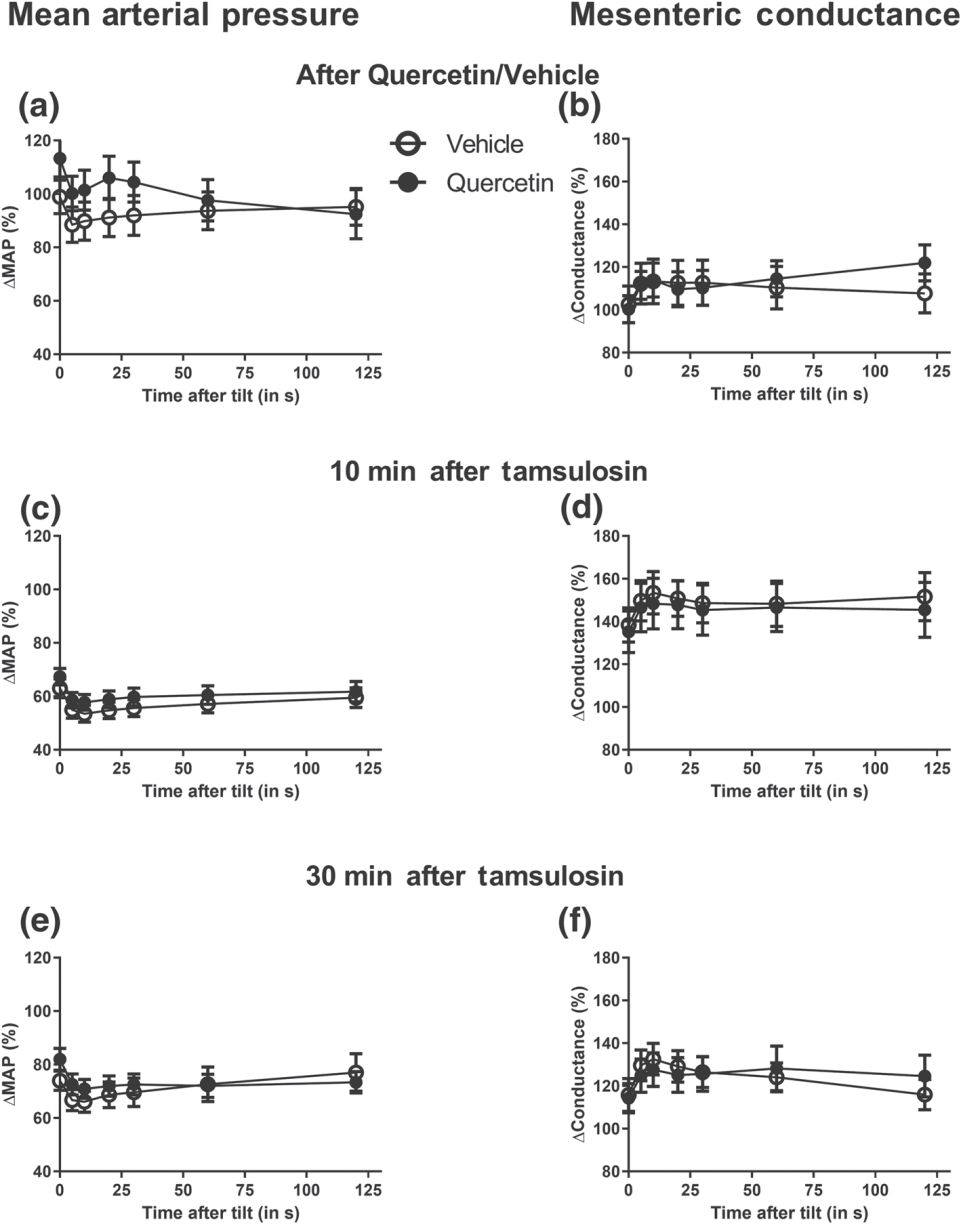
Comparison of the regional haemodynamic changes induced by a 2 min, 45° head‐up tilt in isoflurane anaesthetized Wistar Kyoto rats instrumented with Doppler flow probes. Data are presented for changes in mean arterial pressure (MAP; Figures 4a,c and e) and mesenteric conductance (Figures 4b, d and f) recorded after injection of vehicle or quercetin and hence 10 and 30 min after additional tamsulosin administration. Data are compared for vehicle‐ and quercetin‐treated rats. There was no difference in tilt responses between vehicle‐ and quercetin‐treated rats

Vehicle or quercetin injections did not alter BP, HR or regional (carotid, hindquarter, mesenteric and renal) conductance in anaesthetized WKY rats (Figure 3). Injection of tamsulosin (3 μg·kg^−1^) reduced BP by about 50% and increased regional conductance maximally by 300% in the various vascular beds (Figures 3 and S3). HR remained stable during the interventions. The haemodynamic changes evoked by tamsulosin were nearly back to baseline within 30 min after injection. The duration of the regional vascular effects induced by tamsulosin were not different between vehicle‐ or quercetin‐treated rats. At regular intervals in this protocol, rats were subjected to a 2‐min orthostatic challenge. The orthostatic effects on BP and mesenteric conductance are summarized in Figure 4. As shown in this figure, tilting by 45° caused a rapid 10% decrease in BP, which was associated with a 10% rise in mesenteric conductance. The magnitude of the effect was constant at all time points in the protocol and there was no difference between quercetin‐ and vehicle‐treated rats.

### 3.3 Study 3: BP effects of oral administration of quercetin in SHR equipped with telemetric devices

**Figure 5:**
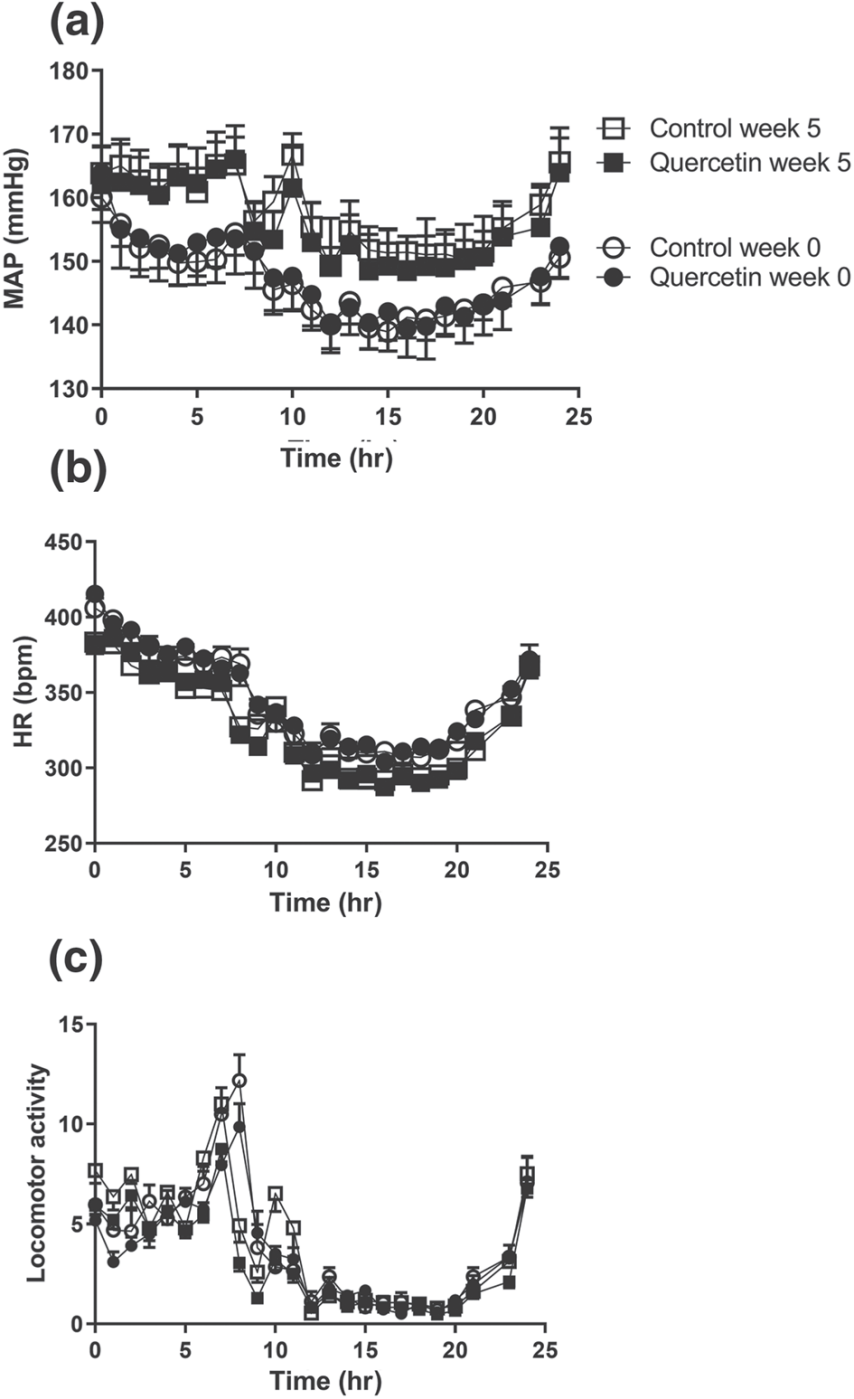
Comparison of telemetry‐based 24‐hr patterns of mean arterial pressure (MAP; Figure 5a) and heart rate (HR; Figure 5b) and locomotor activity (Figure 5c) in adult spontaneously hypertensive rats as recorded over 3 days at the age of 16–17 weeks (defined as week 0) and 5 weeks later (defined as week 5) after chronic oral feeding of quercetin (10 mg·kg^−1^, *n* = 6) or normal food (*n* = 6). In both treatment arms, MAP rose significantly with ageing of the spontaneously hypertensive rats and HR fell. These age‐related changes in haemodynamics were not different between animals on control food and those on quercetin

Figure 5 compares the 24‐hr patterns of BP, HR and locomotor activity in the two groups of SHR designated to receive normal food (control) or quercetin for 5 weeks at 10 mg·kg^−1^·day^−1^. While a considerable variation can be observed in all variables within 24‐hr, none of these data were different between the two treatment groups at this time point (week 0). The oral intake of quercetin did not alter these 24‐hr patterns. In both groups, comparable age‐related increments in BP and reductions in HR were observed.

### 3.4 Study 4: Effects of transient treatment on BP and renal haemodynamics in pre‐hypertensive SHR

The 4‐week supplementation of quercetin to the food of young SHR (4 weeks of age) resulted in a significant growth retardation. As shown in Table 1, at 8 weeks of age, food and water intake was significantly lower when SHR were placed on the food enriched with quercetin. This was associated with significant reductions (about 5–10%) in body weight, organ weight and tibia length. Arterial BP measured in unanaesthetized conditions was lower in quercetin‐supplemented SHR (134 ± 11 mmHg) compared with controls (145 ± 9 mmHg). Renal vascular resistance as determined by direct flow measurements under anaesthesia was not different between groups. When SHR were allowed to eat normal food again for an additional period of 4 weeks, many of the differences that were observed at 8 weeks were normalized except for tibia length and lung weight. Notably, arterial pressures were very comparable at 12 weeks of age (controls: 179 ± 8 mmHg; quercetin‐treated: 180 ± 8 mmHg). Plasma quercetin levels that were reached after 4 weeks of treatment in young SHR were 7.89 ± 1.91 ng·ml^−1^ (*n* = 8), while in control rats (*n* = 7) no quercetin could be detected. It should be noted that these values represent quercetin levels that were present about 5–10 hr after the last ability of the rats to eat. With an approximate plasma half‐life of quercetin of 0.8 hr in rats, estimated plasma levels were probably at least a factor 2^5^ = 32 higher (about 256 ng·ml^−1^, corresponding to 0.8 μmol·L^−1^).

**Table 1:** Metabolic and haemodynamic effects of oral transient feeding of quercetin in pre‐hypertensive spontaneously hypertensive rats from weeks 4 to 8 of age

	Treatment			
Parameters	Control	Quercetin	Control	Quercetin
Age	8 weeks	8 weeks	12 weeks	12 weeks
Observations	*N* = 15	*N* = 12	*N* = 8	*N* = 6
Body weight (g)	167 ± 18	156 ± 11^a^	258 ± 21	239 ± 10
Metabolic parameters				
Urine (ml per 24 hr)	7.4 ± 2.1	6.2 ± 1.7	11.0 ± 1.8	10.3 ± 4.4
Food intake (g per 24 hr)	19.7 ± 2.9	15.9 ± 2.4^a^	23.2 ± 2.1	21.8 ± 3.3
Water intake (ml per 24 hr)	27.0 ± 4.0	21.9 ± 2.6^a^	31.3 ± 4.7	30.0 ± 5.7
Organ weight				
Heart (g)	0.65 ± 0.05	0.62 ± 0.04	1.00 ± 0.08	0.94 ± 0.03
Kidney, left (g)	0.66 ± 0.07	0.61 ± 0.04^a^	0.89 ± 0.06	0.83 ± 0.05
Kidney, right (g)	0.67 ± 0.07	0.62 ± 0.03^a^	0.90 ± 0.06	0.84 ± 0.04
Lungs (g)	0.83 ± 0.08	0.80 ± 0.05	1.10 ± 0.11	0.97 ± 0.05^b^
Tibia length (cm)	3.24 ± 0.08	3.17 ± 0.06^a^	3.71 ± 0.07	3.59 ± 0.03^c^
Cardiovascular variables (unanaesthetized)				
MAP (mmHg)	145 ± 11	134 ± 9^a^	179 ± 8	180 ± 8
HR (bpm)	412 ± 41	396 ± 18	382 ± 26	393 ± 26
RBF measurement (anaesthesia)				
MAP (mmHg)	93 ± 17	88 ± 17	116 ± 40	112 ± 16
RBF (ml·min^−1^ per 100 g KW)	3.39 ± 1.01	3.64 ± 0.94	3.41 ± 1.05	3.21 ± 0.66
Conductance (units)	0.04 ± 0.01	0.04 ± 0.01	0.03 ± 0.02	0.03 ± 0.01

*Note.* In a subgroup of spontaneously hypertensive rats, after the 4‐week treatment period, quercetin was withdrawn and replaced by normal food. At the age of 12 weeks, the same measurements were performed.

Abbreviations: HR, heart rate; KW, kidney weight; MAP, mean arterial pressure; RBF, renal blood flow.

a Compared with control, 8 weeks, *P* < .05.

b Compared with control, 12 weeks, *P* < .05.

c Compared with control, 12 weeks, *P* < .01.

## 4 DISCUSSION

The present study revisited the *in vivo* haemodynamic effects of quercetin in rats. Remarkably, all of the measurements indicated that quercetin does not lower arterial BP *in vivo*, either after acute intravenous administration or after chronic oral feeding. Moreover, quercetin did not affect the haemodynamics of anaesthetized rats that were exposed to an orthostatic challenge. Hence, the study failed to translate the recent *in vitro* observation showing an interaction between quercetin and tamsulosin to the *in vivo* situation (Vrolijk et al., 2015).

Reports on the BP‐lowering action of quercetin in humans are not univocal. In the most recent systematic review and meta‐analysis, a small (−3 mmHg) effect was found (Serban et al., 2016). Significant BP reductions occurred only when doses of quercetin exceeded 500 mg·day^−1^. For doses <500 mg·day^−1^, the size of the effect was no different from placebo. In most experimental animal studies, modest BP reductions by quercetin have been reported (Duarte et al., 2001; Jalili et al., 2006). Only in one rat study, quercetin failed to lower BP (Carlstrom et al., 2007). To our knowledge, other negative studies have not been published. Mechanisms and the magnitude and relevance of the antihypertensive effects of quercetin remain unclear. In rat studies, dosing of quercetin is usually well controlled and above 5 mg·kg^−1^·day^−1^. Therefore, differences in BP‐lowering efficacy of quercetin have not been attributed to the dose of the nutraceutical but rather to other factors such as (a) the age of the rats, (b) the type of model and severity of hypertension, (c) the particular form of quercetin administered and (d) the route of delivery of the agent (Carlstrom et al., 2007). The present study combines data from four sub‐studies on the haemodynamic effects of quercetin. The whole data set embraces many aspects of the four factors mentioned above and includes acute and chronic effects of quercetin, being orally and intravenously administered, in unanaesthetized and anaesthetized conditions and in pre‐hypertensive and normotensive rats.

4.1

#### 4.1.1. Study 1: Does quercetin amplify the α_1_‐adrenoceptor antagonist properties of tamsulosin *in vivo*?

Acute intravenous administration of quercetin did not modify α_1_‐receptor‐mediated changes in haemodynamics evoked by phenylephrine in conscious SHR and WKY rats. This indicates that quercetin has no interference with vascular α_1_‐receptors *in vivo.* The food additive did also not augment tamsulosin‐mediated reductions in arterial BP. While the tamsulosin‐induced fall in BP was larger in SHR than in WKY rats, the presence of quercetin did not reduce BP any further, excluding a pharmacodynamic interaction. Since quercetin did not modify the duration of the BP‐lowering effect in either, a kinetic interaction between tamsulosin and quercetin is unlikely. It has been suggested that quercetin might modify the baroreflex‐mediated changes in HR (Monteiro et al., 2012). However, the present data do not support this suggestion since the HR increments that occurred in response to the tamsulosin‐induced fall in arterial pressure were not different between quercetin‐ and vehicle‐treated rats. The magnitude of the haemodynamic responses to quercetin and tamsulosin was also compared between male and female rats. Sex differences were not detected in either protocols (data not shown). Thus, while quercetin increased the potency of tamsulosin in mesenteric arteries *in vitro* (Vrolijk et al., 2015), such an effect could not be reproduced *in vivo.*

#### 4.1.2. Study 2: Regional vascular effects of quercetin in the absence and presence of tamsulosin

Although quercetin did not amplify tamsulosin‐induced systemic reductions in arterial BP, this finding does not exclude that quercetin might modify regional vascular responses to the α_1_‐antagonist in a differential way. To examine this possibility, regional vascular responses to tamsulosin were compared in the presence and absence of quercetin. Attention was paid particularly to responses that occurred in the mesenteric vascular bed, because it was the arteries of this organ that the *in vitro* interaction between tamsulosin and quercetin was originally observed (Vrolijk et al., 2015). The data showed that injection of tamsulosin in anaesthetized WKY rats reduced BP rapidly (within 3 min) by more than 40 mmHg and that this was caused by an increase in vascular conductance (dilation) in all four of the vascular beds being monitored. The duration of these increases were found to be unchanged quercetin treatment. These observations also exclude a regional vascular preference for the observed *in vitro* interaction between the drug and the food additive. To investigate if quercetin could augment tamsulosin‐related orthostatic haemodynamic responses, rats were challenged to a 45° head‐up tilt. While this was associated with a 5‐ to 10‐mmHg decreases in arterial BP and increases in mesenteric conductance, the magnitude of the responses to tilting was not different between vehicle‐ and quercetin‐treated rats. These combined present observations in rats do not support the hypothesis that quercetin may aggravate orthostatic challenges by tamsulosin in patients. It has been proposed that tamsulosin has already a low tendency to cause orthostasis in humans because of its specific binding properties to the plasma protein alpha‐1‐glycoprotein (Noble et al., 1997). Because quercetin binds predominantly to plasma albumin (Diniz et al., 2008) and not alpha‐1‐glycoprotein, such a kinetic interaction is also not likely. Alternatively, it is possible that *in vivo* flow‐mediated endothelial factors may have overruled the *ex vivo* interaction between tamsulosin and quercetin. Using *in vivo* imaging techniques, it was found that 7‐mono‐*O*‐(β‐hydroxyethyl)‐rutoside, a semi‐synthetic derivative of quercetin, was present inside the endothelial cells of intact arteries (Lemmens et al., 2014). The significance of this observation needs further exploration.

#### 4.1.3. Study 3: BP effects of oral administration of quercetin in SHR equipped with telemetric devices

Significant antihypertensive properties for quercetin have been mainly reported in rat studies in which BP was measured indirectly by tail cuff plethysmography. This procedure has the limitation in that the animals need to be warmed and restrained and provides systolic pressure readings over a few cardiac cycles that are not stress free (Kurtz et al., 2005). Typically, in such studies in SHR, systolic pressures >220 mmHg were noted and quercetin‐induced reductions in systolic pressures were in the order of 5–20%. In contrast, in telemetry studies, rats are housed in their home cage and data are collected over a full 24‐hr period under stress‐free conditions. Using the latter approach, 24‐hr BP profiles were compared in adult SHR before and after a 5‐week treatment with quercetin. In both the control‐ and quercetin‐treated rats, BP rose by about 15 mmHg between weeks 16 and 22 of age. At no point in time over this measurement period, a BP‐lowering effect of quercetin was observed. Because 24‐hr profiles of HR and locomotor activity were also very comparable, the data suggest that quercetin treatment did also not influence behavioural activity of the rats. To our knowledge, this is the first telemetric study that reports the absence of BP‐lowering action of quercetin.

#### 4.1.4. Study 4: Effects of transient treatment on BP and renal haemodynamics in prehypertensive SHR

While the addition of 0.1% quercetin through the regular rat chow led to biologically effective plasma concentrations of quercetin (Boots et al., 2011), the treatment regimen was associated with significant reductions in food and water intake in young pre‐hypertensive SHR. Probably, the young rats disliked the taste of the quercetin aglycone as significant growth retardation occurred. Hence, the lower arterial BP that was found after 4 weeks of ingesting quercetin should not be attributed to a direct effect of the food additive but should rather be interpreted as being a consequence of the retarded general development of the SHR, thereby delaying the progression of high BP in this strain. This explanation is supported by the observation that when normal food was allowed for an additional period of 4 weeks, catch‐up growth occurred and BP rose to levels as observed in untreated SHR. In pre‐hypertensive SHR, the renal vascular bed is among one of the vascular beds that exhibit reduced vascular conductance over a wide vasomotor range (Evenwel, Kasbergen, & Struyker‐Boudier, 1983; Kett, Bergstrom, Alcorn, Bertram, & Anderson, 2001). For this reason, renal vascular conductance was compared by direct blood flow measurements in SHR receiving quercetin enriched or control food. Renal vascular resistance was not different between these treatments. Heijnen et al. (2014) and Baumann et al. (2007) showed that pre‐hypertensive treatment with an angiotensin receptor antagonist in the same developmental period in SHR induces a long‐term antihypertensive effect. In the current study, no such effects were found for quercetin. Potentially, quercetin‐related effects are more evident in conditions of high renin–angiotensin–aldosterone system activity, such as in rats in which the abdominal aorta was clipped suprarenally (Jalili et al., 2006).

Overall, after extensively investigation of the effects of quercetin on the haemodynamics of rats, our results show that quercetin administration does not affect haemodynamics in both SHR and WKY rats. The literature suggests that the mode of administration may be important in determining the protective effects of quercetin (Carlstrom et al., 2007) and that beneficial effects may be mediated by metabolites produced by gut microbiota (Santangelo, Silvestrini, & Mancuso, 2019). The present study was not designed to examine such potential explanations. We administered the most common form of quercetin in doses that would be present in the human diet or when taken as food supplement.

In summary, different modes of delivery of quercetin, different exposure times, different ages of the rats and different approaches were used to study haemodynamic effects of quercetin in the present study. Not only is the interaction between quercetin and tamsulosin investigated *in vivo* for the first time, but also telemetry and the tilt table were used for the first time to study haemodynamic effects of quercetin in rats. We have shown that the *in vitro* discovered interaction between quercetin and tamsulosin could not be validated *in vivo.*

## AUTHOR CONTRIBUTIONS

M.F.V., A.B., and B.J.J. conceived and designed the studies. H.v.E. and B.J.J. conducted the experiments. M.F.V., H.v.E., and B.J.J. performed the data analysis. M.F.V., A.O., A.B., and B.J.J. interpreted the data. M.F.V. and B.J.J. wrote the original draft. H.v.E., A.O., and A.B. reviewed and revised the manuscript for publication.

## CONFLICT OF INTEREST

The authors declare no conflicts of interest.

## DECLARATION OF TRANSPARENCY AND SCIENTIFIC RIGOUR

This Declaration acknowledges that this paper adheres to the principles for transparent reporting and scientific rigour of preclinical research as stated in the *BJP* guidelines for https://bpspubs.onlinelibrary.wiley.com/doi/full/10.1111/bph.14207 and https://bpspubs.onlinelibrary.wiley.com/doi/full/10.1111/bph.14206 and as recommended by funding agencies, publishers, and other organizations engaged with supporting research.

## Supporting information


**Figure S1**: Baseline values of bodyweights, mean arterial pressure, and heart rate between vehicle‐treated and quercetin treated normotensive Wistar Kyoto rats (WKY, *n* = 8) and spontaneously hypertensive rats (SHR, *n* = 8) as obtained in protocol 1A and protocol 1B. Haemodynamic values are presented as averages recorded over a 30‐minute baseline period in the conscious state. There was no effect of treatment of quercetin on any of these parameters in WKY or SHR.

**Figure S2**: Drawing of the laboratory setup of the regional flow measurements during the tilt studies under anaesthetised conditions. The table can be manually returned in the horizontal position without changing the level of pressure transducer, fixed at the level of the heart.

**Figure S3**: Screen dump of an original tracing showing the acute regional haemodynamic effects of tamsulosin (3 μg.kg^−1^ i.v.) as well as two periods of 2 minutes 45 degrees head‐up tilt in isoflurane anaesthetized Wistar Kyoto rats instrumented with Doppler flow probes. Data are presented as beat‐to‐beat changes in systolic (yellow) and diastolic (red) values of mean arterial blood pressure (BP) and heart rate (HR), hind quarter blood flow (HQBF), mesenteric blood flow (MBF), carotid blood flow (CaBF) and renal blood flow. (RBF) over a 16 minutes period. In this example, during tilting, the Doppler flow signal of the renal probe (RBF) was lost due to the change in position of the rat. Such artefacts were excluded from analysis.
