# Supplementary figures and images for: Haemodynamic effects of the flavonoid quercetin in rats revisited
Source: Br J Pharmacol. 2020 Feb 3;177(8):1841–52. doi: 10.1111/bph.14955 (PMC7070173; doi:10.1111/bph.14955)

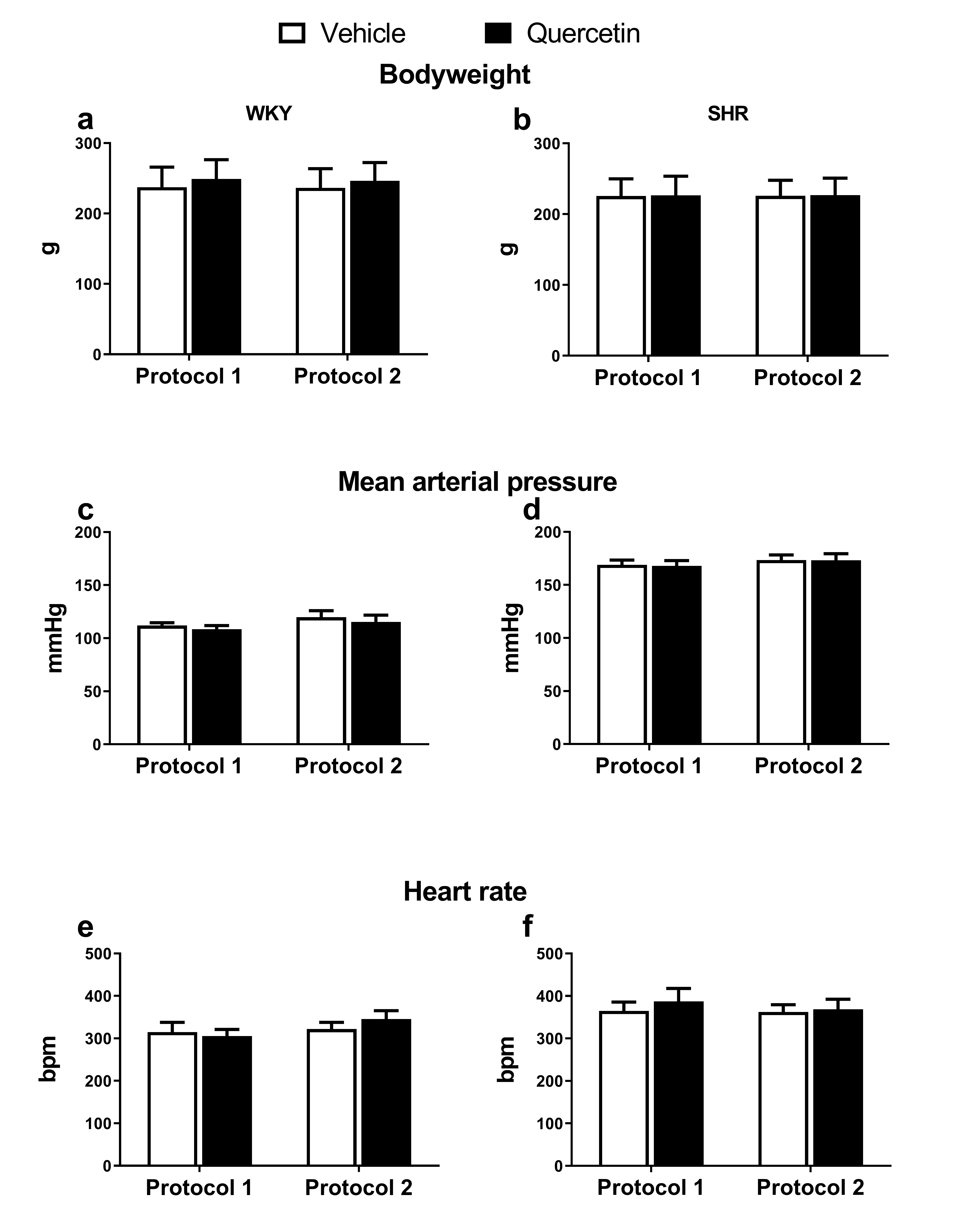

Supplement: Supplementary file 1 — Figure S1: Baseline values of bodyweights, mean arterial pressure, and heart rate between vehicle‐treated and quercetin treated normotensive Wistar Kyoto rats (WKY, n = 8) and spontaneously hypertensive rats (SHR, n = 8) as obtained in protocol 1A and protocol 1B. Haemodynamic values are presented as averages recorded over a 30‐minute baseline period in the conscious state. There was no effect of treatment of quercetin on any of these parameters in WKY or SHR. [file BPH-177-1841-s001.tif]

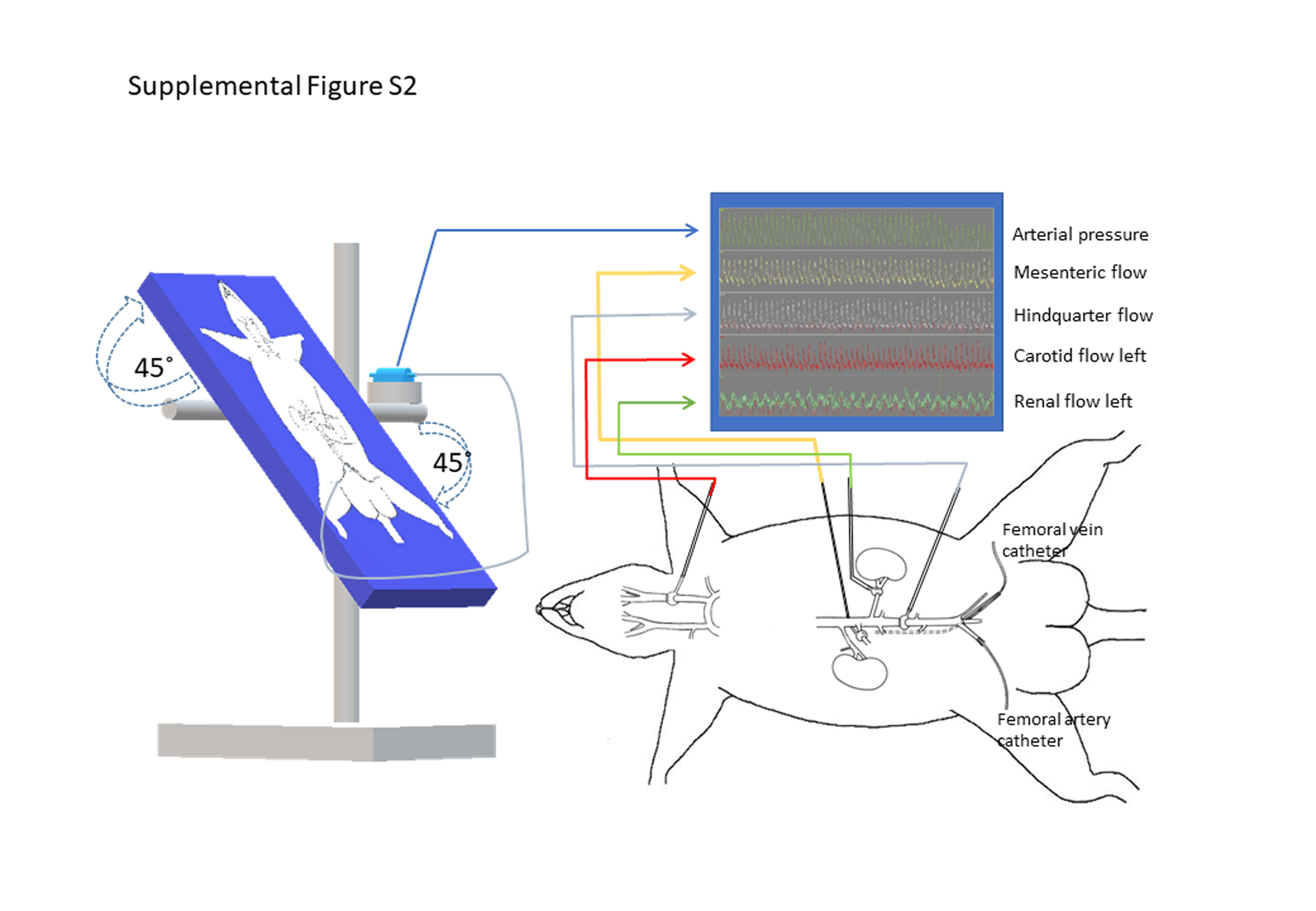

Supplement: Supplementary file 2 — Figure S2: Drawing of the laboratory setup of the regional flow measurements during the tilt studies under anaesthetised conditions. The table can be manually returned in the horizontal position without changing the level of pressure transducer, fixed at the level of the heart. [file BPH-177-1841-s002.tif]

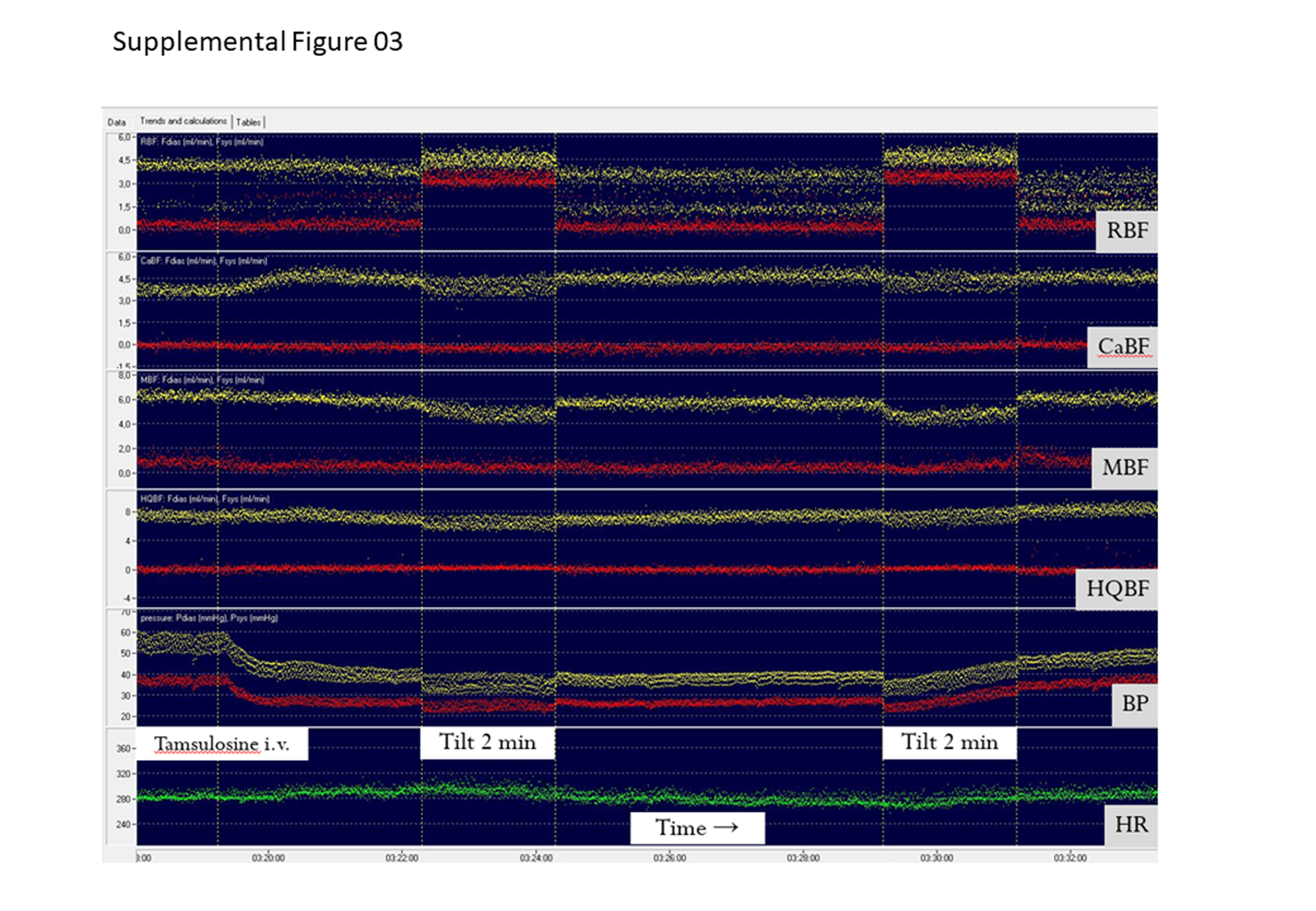

Supplement: Supplementary file 3 — Figure S3: Screen dump of an original tracing showing the acute regional haemodynamic effects of tamsulosin (3 μg.kg−1 i.v.) as well as two periods of 2 minutes 45 degrees head‐up tilt in isoflurane anaesthetized Wistar Kyoto rats instrumented with Doppler flow probes. Data are presented as beat‐to‐beat changes in systolic (yellow) and diastolic (red) values of mean arterial blood pressure (BP) and heart rate (HR), hind quarter blood flow (HQBF), mesenteric blood flow (MBF), carotid blood flow (CaBF) and renal blood flow. (RBF) over a 16 minutes period. In this example, during tilting, the Doppler flow signal of the renal probe (RBF) was lost due to the change in position of the rat. Such artefacts were excluded from analysis. [file BPH-177-1841-s003.tif]
